# Measuring and Estimating GFR and Treatment Effect in ADPKD Patients: Results and Implications of a Longitudinal Cohort Study

**DOI:** 10.1371/journal.pone.0032533

**Published:** 2012-02-28

**Authors:** Piero Ruggenenti, Flavio Gaspari, Antonio Cannata, Fabiola Carrara, Claudia Cella, Silvia Ferrari, Nadia Stucchi, Silvia Prandini, Bogdan Ene-Iordache, Olimpia Diadei, Norberto Perico, Patrizia Ondei, Antonio Pisani, Erasmo Buongiorno, Piergiorgio Messa, Mauro Dugo, Giuseppe Remuzzi, for the GFR-ADPKD Study Group

**Affiliations:** 1 Clinical Research Center for Rare Diseases Aldo & Cele Daccò, Mario Negri Institute for Pharmacological Research, Bergamo, Italy; 2 Unit of Nephrology, Azienda Ospedaliera Ospedali Riuniti di Bergamo, Bergamo, Italy; 3 Azienda Ospedaliera Universitaria Federico II, Napoli, Italy; 4 Presidio Ospedaliero V. Fazzi, Lecce, Italy; 5 Fondazione IRCCS Ca' Granda Ospedale Maggiore Policlinico, Milano, Italy; 6 Azienda ULSS 9 – Ospedale S. Maria di Ca' Foncello, Treviso, Italy; INSERM, France

## Abstract

Trials failed to demonstrate protective effects of investigational treatments on glomerular filtration rate (GFR) reduction in Autosomal Dominant Polycystic Kidney Disease (ADPKD). To assess whether above findings were explained by unreliable GFR estimates, in this academic study we compared GFR values centrally measured by iohexol plasma clearance with corresponding values estimated by Chronic Kidney Disease Epidemiology Collaboration (CKD-Epi) and abbreviated Modification of Diet in Renal Disease (aMDRD) formulas in ADPKD patients retrieved from four clinical trials run by a Clinical Research Center and five Nephrology Units in Italy. Measured baseline GFRs and one-year GFR changes averaged 78.6±26.7 and 8.4±10.3 mL/min/1.73 m^2^ in 111 and 71 ADPKD patients, respectively. CKD-Epi significantly overestimated and aMDRD underestimated baseline GFRs. Less than half estimates deviated by <10% from measured values. One-year estimated GFR changes did not detect measured changes. Both formulas underestimated GFR changes by 50%. Less than 9% of estimates deviated <10% from measured changes. Extent of deviations even exceeded that of measured one-year GFR changes. In ADPKD, prediction formulas unreliably estimate actual GFR values and fail to detect their changes over time. Direct kidney function measurements by appropriate techniques are needed to adequately evaluate treatment effects in clinics and research.

## Introduction

Seven to ten percent of patients requiring chronic renal replacement therapy because of end-stage renal disease (ESRD) are affected by Autosomal Dominant Polycystic Kidney Disease (ADPKD) [1]–[3]. Renal function loss in ADPKD is largely related to the development and growth of cysts and concomitant disruption of normal renal tissue [4]. Thus, experimental and clinical studies tested drugs that specifically target factors - such as cyclic AMP and mammalian Target of Rapamycin (mTOR) related pathways [5], [6] - that appear to be involved in the dysregulation of epithelial cell growth, secretion, and matrix deposition that is characteristic of the disease [7]–[11]. The enthusiasm on this line of research, however, was stifled by the results of recent trials showing no appreciable protective effect of sirolimus or everolimus therapy against progressive glomerular filtration rate (GFR) decline in two large cohorts of ADPKD patients [12], [13]. In both trials the GFR was estimated (eGFR) by using prediction formulas - the “Chronic Kidney Disease Epidemiology Collaboration” (CKD-Epi) and the “abbreviated Modification of Diet in Renal Disease” (aMDRD) equations – that are based on serum creatinine level, taken as an endogenous marker of glomerular filtration [Levey A, et al. (2000) J Am Soc Nephrol 11: 155A, Abstract] [14]. These formulas, however, has been repeatedly challenged and there is increasing evidence that their use might generate misleading information in particular in subjects with normal or near normal kidney function [15]–[19] [Porrini E et al. (2010) American Society of Nephrology, Renal Week 2010, Denver, CO, November 16–21, Abstract F-PO1244]. Thus, direct measurements of the GFR by gold-standard techniques based on the use of exogenous markers of glomerular filtration such as inulin, iohexol or radio-labeled tracers [20]–[24] would be needed to adequately assess a treatment effect on GFR decline in this population. To test this hypothesis we took advantage from a cohort of ADPKD patients prospectively monitored by serial GFR measurements and estimations in the setting of controlled clinical trials coordinated by the Mario Negri Institute for Pharmacological Research in Italy. In this population we evaluated the relationships between GFR values centrally measured (mGFR) at inclusion and at one-year follow-up and the concomitant GFR estimates obtained by prediction formulas (eGFR). To this purpose the GFR was measured by using the iohexol plasma clearance technique [21], a procedure previously validated by comparative analyses with inulin renal clearance showing that iohexol is a reliable marker of glomerular filtration in normal subjects as well as in patients with different degree of renal insufficiency [21]. Compared to renal inulin clearance this procedure does not require urine collection or continuous infusion of the filtration marker, and compared to ^51^Cr-EDTA and ^99m^Tc-DTPA plasma clearance techniques [25], [26], it allows avoiding the use of radiolabeled tracers. Both advantages facilitate kidney function monitoring in everyday clinical practice and in research [21], [27]–[29]. Thus, the availability of direct GFR measurements allowed to test the reliability of prediction formulas in patients with ADPKD and to assess whether and to which extent their use can affect the statistical power of a clinical trial aimed to detect the protective effect of a given intervention on progressive renal function loss in this population.

## Methods

This was a fully academic, internally funded study with no sponsor or company involvement in study design and data recording, analysis, interpretation and reporting. All considered trials conformed to the Declaration of Helsinki guidelines and were approved by the “Comitato di Bioetica della Azienda Ospedaliera Ospedali Riuniti di Bergamo”. In addition, for the ALADIN Study Ethics Committees of Lecce, Milan, Naples, and Treviso approved the trial. All included patients provided written consent to trial participation. Data were handled in respect of patient anonymity and confidentiality.

### Study population

We used measured and estimated GFR data obtained from homogeneous cohorts of adult ADPKD subjects with baseline eGFR >30 mL/min/1.73 m^2^ (by aMDRD equation) who had been included in four studies designed, conducted, and monitored by the Investigators of the Clinical Research Center for Rare Diseases *“Aldo e Cele Daccò”* of the Mario Negri Institute (Bergamo, Italy). The “Safety and Efficacy of Long-acting Somatostatin Treatment in Autosomal Dominant Polycystic Kidney Disease” study [30] and the “Sirolimus Treatment in Patients with Autosomal Dominant Polycystic Kidney Disease” I (SIRENA I, EUDRACT N°: 2006-003427-37) [31] and II (SIRENA II, EUDRACT N°: 2007-005047-21) studies were run in cooperation with the Nephrology Unit in Bergamo, whereas the “Effects of Long-acting Somatostatin on Disease Progression in Patients with Autosomal Dominant Polycystic Kidney Disease and Moderate to Severe Renal Insufficiency Therapy” (ALADIN, EUDRACT N°: 2005-005552-41) study involved also four Units in Lecce, Milan, Naples, and Treviso, all in Italy. The first two studies evaluated the short-term effects of six-month therapy with Sandostatin-LAR® Depot (Novartis Farma S.p.A., Origgio, Varese, Italy) or Rapamune® (Wyeth-Lederle S.p.A., Aprilia, Latina, Italy) on kidney volumes and function in the setting of a randomized, cross-over design. The other two studies evaluated the long-term effect of three-year treatment with the two agents on kidney volumes and mGFR decline in the setting of a randomized, parallel-group design. Baseline data were obtained from all studies (Somatostatin study: 10, SIRENA I: 21, SIRENA II: 4, ALADIN: 76), whereas one-year data were available only from the ALADIN study.

All studies excluded ADPKD patients with evidence of concomitant systemic, renal parenchymal (proteinuria ≥1 gr/24 hours) or urinary tract disease, diabetes, cancer, psychiatric disorders, as well as pregnant or lactating women or fertile women without effective contraception. Considered variables were recorded according to similar timetables in case record forms and databases which had a similar frame. Thus, the homogeneity in patient characteristics, study design and organization, monitored variables, and data handling procedures allowed the merging of data in a common meta-database and all considered outcomes could be analyzed with a minimized risk of reasonably predictable biases.

### GFR measurement and estimation

GFR was centrally determined at the laboratory of the Clinical Research Center at patient inclusion and one year apart by using the iohexol plasma clearance technique. GFR was determined by the plasma clearance of iohexol. Briefly, on the morning of renal function evaluation, 5 ml of iohexol solution (Omnipaque 300, GE Healthcare, Milan, Italy) was injected intravenously over 2 minutes. Blood samples were then taken at 120, 180, 240, 300, 360, 420, and 480 min for patients with expected mGFR≤40 mL/min, and at 120, 150, 180, 210, and 240 min for those with expected mGFR>40 mL/min. Blood iohexol plasma levels were measured by high-performance-liquid chromatography. The clearance of iohexol was calculated according to a one-compartment model (CL_1_) by the formula: CL_1_ = Dose/AUC, where AUC is the area under the plasma concentration-time curve. According to Bröchner-Mortensen [32], plasma clearances were then corrected by using the formula CL = (0.990778×CL_1_)−(0.001218×CL_1_^2^). GFR values were then normalized to 1.73 m^2^ of body surface area (BSA).

The procedure has remarkable precision over a wide range of kidney function [33] as documented by the low mean intra-individual coefficient of variation (5.59%) and good reproducibility index (6.28%) observed in repeated measurements in subjects with near-terminal kidney failure, normal GFR or even hyperfiltration.

The morning of each iohexol clearance study, serum creatinine concentration was measured with the modified rate Jaffé method using an automatic device (Beckman Synchron LX20 Pro, Beckman Coulter S.p.A., Cassina De' Pecchi, Italy) and demographic and anthropometric data considered in CKD-Epi and aMDRD equations [Levey A, et al. (2000) J Am Soc Nephrol 11: 155A, Abstract] [14] were recorded. For CKD-Epi estimates, measured serum creatinine values were standardized to the isotope-dilution-mass-spectrometry method by the equation provided by Beckman-Coulter. GFR values estimated by both models were normalized.

### Statistical analysis

Data were expressed as mean ± standard deviation (SD) or median. The relationships between measured and estimated GFR values at baseline, as well as between one-year changes in measured and estimated GFRs, were studied by regression analyses considering Pearson correlation coefficient and Lin concordance correlation coefficient as an index to evaluate the degree to which pairs of observations fall on the 45° line through the origin [34]. Additional sensitivity analyses were performed by using the Deming regression. Analyses were performed in the study group as a whole and in two subgroups with baseline mGFR≥ or <70 mL/min/1.73 m^2^ considered separately.

Bias, mean percent error (MPE) and mean percent absolute error (MAPE) were determined as previously described [35]. Scatter was defined as the median absolute difference between measured and estimated GFR. Taking into account that the reproducibility of the iohexol plasma clearance is 6.28% [33], the eGFR values lying within the ±10% error range were a priori considered as virtually identical to mGFR values. The trend of the errors was represented by Bland-Altman analysis: the differences between estimated and measured GFRs (or estimated and measured one-year GFR differences) were plotted versus the mean of estimated and measured GFRs (or estimated and measured one-year GFR differences). Data were compared by paired or unpaired t-test, Mann-Whitney test, chi-square test or one way analysis of variance (ANOVA), as appropriate. The statistical significance level was defined as p<0.05. All analyses were performed by MedCalc (11.3.3 version) or MS Excel.

## Results

### Patient characteristics

Baseline data were available from 111 patients. They were relatively young and predominantly male subjects (Table 1). Thirty-three patients were overweight and 13 obese (according to a body mass index between 25 and 30 kg/m^2^ or exceeding 30 kg/m^2^, respectively). Serum creatinine exceeded the upper limit of the normal range (1.30 mg/dL) in 39 cases. The GFR was less than the lower limit of the normal range (80–120 mL/min/1.73 m^2^) in 62 cases. Only six patients were hyperfiltering (mGFR>120 mL/min/1.73 m^2^). No patient was on concomitant treatment with drugs known to interfere with creatinine tubular handling.

**Table 1:** Patients characteristics at inclusion.

	Whole study group	Patients with baseline and one-year data	Patients with baseline data only
**n**	111	71	40
**Age (yr)**	38.20±7.87	37.15±7.98	40.06±7.40
**Male sex – no. (%)**	62(55.86)	34(47.89)	28(70.00)
**Height (cm)**	171.12±9.50	170.01±9.85	173.08±8.62
**Weight (Kg)**	73.28±14.65	72.49±14.58	74.68±14.86
**Body Mass Index (kg/m^2^)** †	24.91±3.86	24.96±3.91	24.81±3.83
**Body Surface Area (m^2^)** $	1.85±0.21	1.83±0.21	1.88±0.21
**Systolic Blood Pressure (mmHg)**	130.21±15.50	127.74±15.49	136.06±14.08∧
**Diastolic Blood Pressure (mmHg)**	84.91±11.22	84.07±12.22	86.89±8.25
**Serum creatinine (mg/dL)** ∫	1.21±0.46	1.12±0.45	1.37±0.46∧ *
**Uric Acid (mg/dL)**	5.41±1.61	5.18±1.63	5.83±1.50∧
**GOT/AST (U/L)**	19.99±6.36	19.61±7.24	20.69±4.33
**GPT/ALT (U/L)**	20.09±11.07	19.48±12.39	21.21±8.17
**Proteinuria (g/24 h)**	0.25±0.52	0.28±0.62	0.16±0.13
**Albuminuria (µg/min)**	52.86±65.02	61.89±79.63	40.81±35.36
**GFR (mL/min/1.73 m^2^)**	78.56±26.70	83.13±27.52	70.45±23.38∧
**Antihypertensive therapy - no. (%)**	73 (65.77)	44 (61.97)	29 (72.50)
**ACEi – no. (%)**	55 (49.55)	33 (46.48)	23 (57.50)
**CCB – no. (%)**	12 (10.81)	8 (11.27)	4 (10.00)
**ARBs – no. (%)**	23 (20.72)	16 (22.54)	7 (17.50)
**Antihypertensive drugs - no.**	2 (1–3)	2 (1–3)	1 (1–2)

Data are mean±SD.

† The body mass index is the weight in kilograms divided by the square of the height in meters.

$ The body surface area is calculated with the Dubois&Dubois formula.

∫ To convert values for serum creatinine to micromoles per liter, multiply by 88.4.

* p<0.05 vs. whole study group;

∧ p<0.05 vs. patients with baseline and one-year data.

### Relationships between measured and estimated GFR at baseline

**Figure 1:**
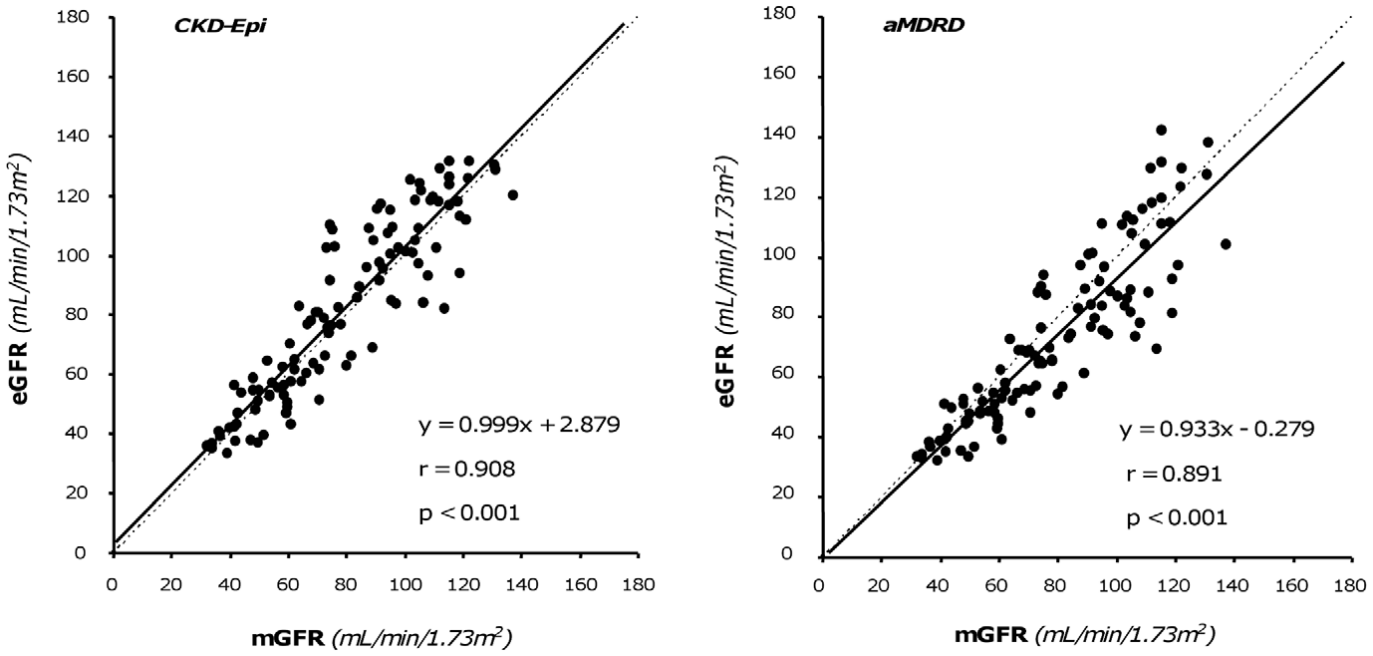
Correlation between estimated and measured by iohexol plasma clearance GFR. Values estimated by CKD-Epi and aMDRD formulas are shown in the left and right panel respectively. Dot lines are identity lines; continuous lines are regression lines.

**Figure 2:**
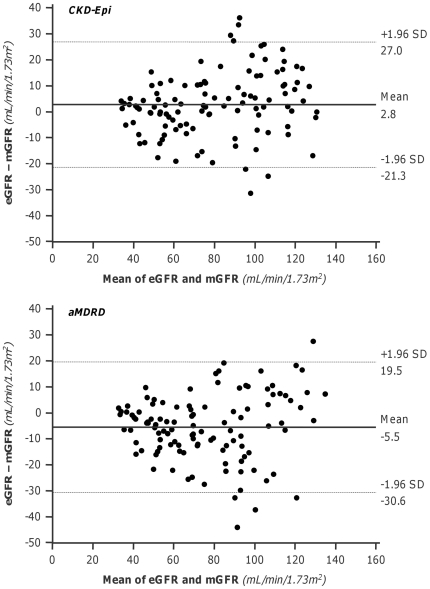
Agreement between measured and estimated GFR values. Bland-Altman plot of the difference between GFR estimated (eGFR) by the CKD-Epi (Upper panel) and by aMDRD (Lower panel) formulas and measured GFR (mGFR) vs. the mean of the two determinations. Straight line and dashed lines indicate mean difference and 95% limits of agreement, respectively.

**Figure 3:**
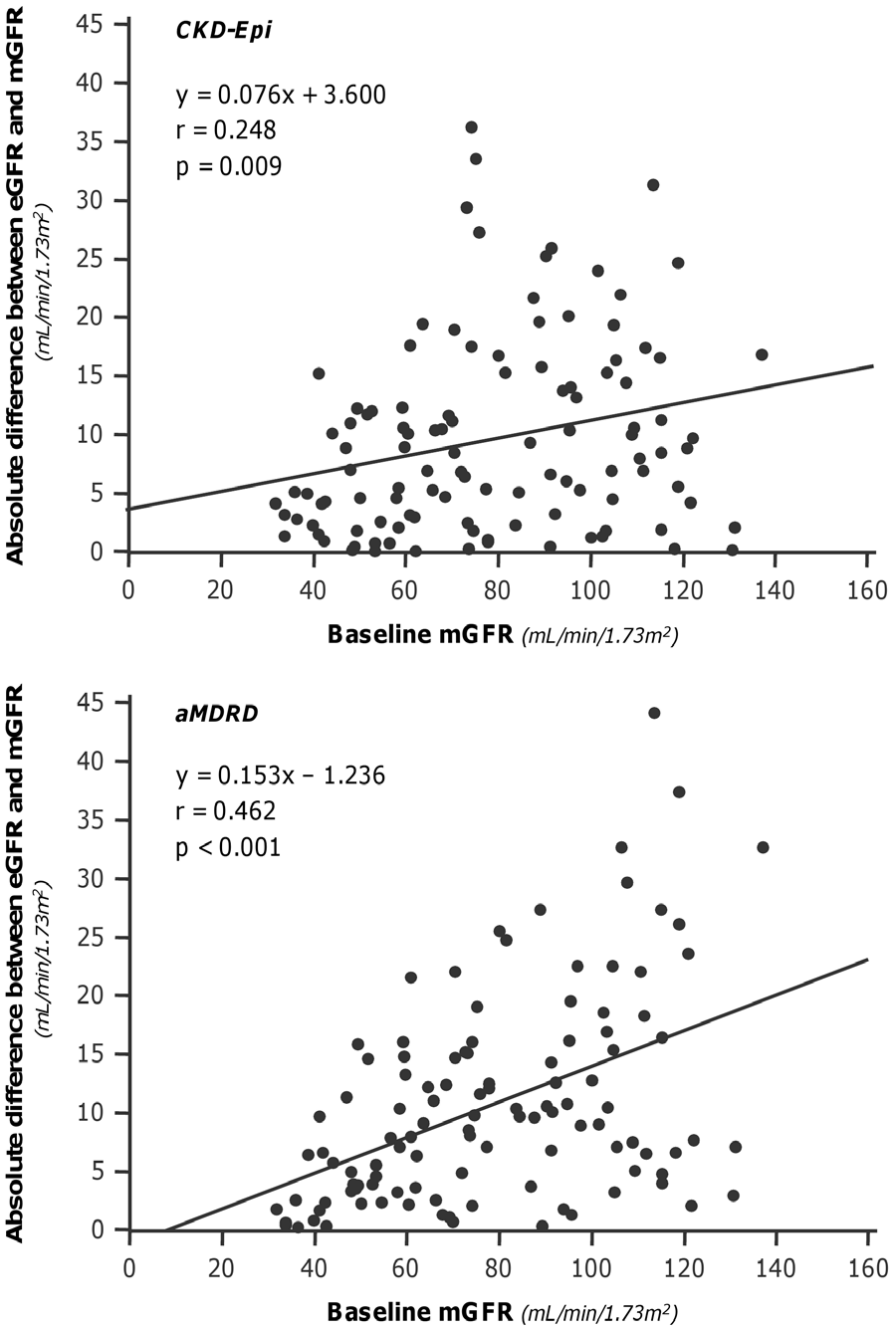
Absolute differences between measured and estimated GFR values vs baseline measured GFR. The absolute differences significantly increase for both CKD-Epi (Upper panel) and aMDRD (Lower panel) formulas for increasing values of GFR. Continuous lines are regression lines.

**Figure 4:**
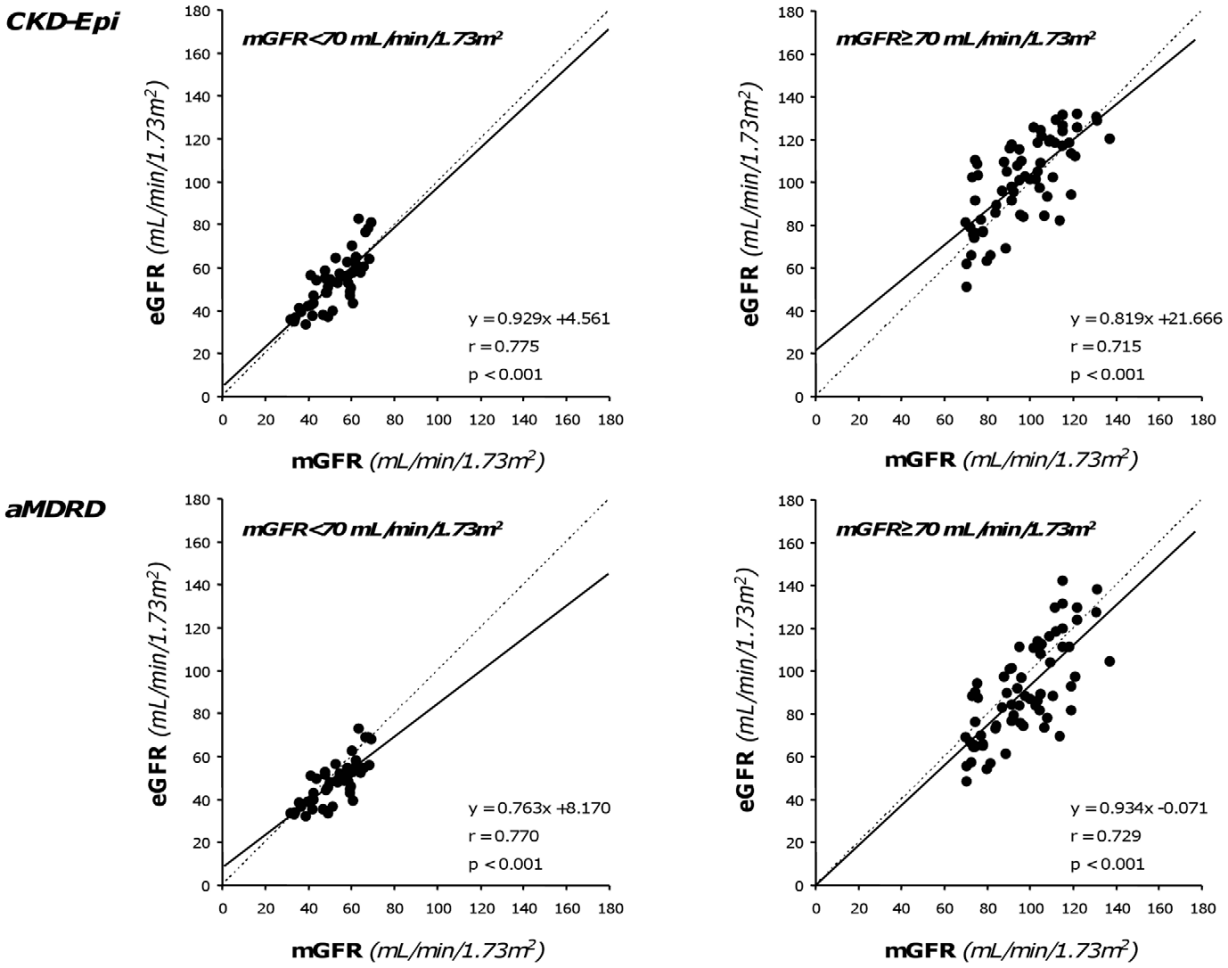
Relationship between GFR values ranked according to renal function. Correlation between GFR measured by iohexol plasma clearance and GFR estimated by the CKD-Epi (Upper panels) and aMDRD (Lower panels) formulas in patients with baseline GFR< or ≥70 mL/min/1.73 m^2^ considered separately (Left and Right panels, respectively). Dot lines are identity lines; continuous lines are regression lines.

The GFRs estimated by CKD-Epi and aMDRD formulas were significantly correlated (p<0.001) with measured GFRs (Figure 1, Left and Right Panel, respectively). The “r” correlation (0.908 vs. 0.891) and Lin concordance (0.899 vs. 0.872) coefficient were slightly higher with CKD-Epi than aMDRD estimates. Similar results were obtained by using the Deming regression model. Analyses indicated a proportional difference (slope statistically different from 1) and a constant negative difference (intercept significantly different from 0) for the CKD-Epi and aMDRD formulas respectively. CKD-Epi significantly overestimated and aMDRD underestimated mGFR values, respectively (Table 2). Mean percent errors vs. actual values showed similar trends, whereas mean absolute percent errors were similar with the two estimates (Table 2). Overall, less than half of the estimates deviated by <10% from actual values. The accuracy was poor for both estimates, although the percentage of acceptable estimates was slightly higher with CKD-Epi than aMDRD. With both formulas, scatter and mean absolute differences between measured and estimated GFR changes ranged between 7 and 11 mL/min/1.73 m^2^. On the basis of the results of Bland-Altman analyses, the performance of the two equations was similarly poor at any degree of renal function, with a trend to greater errors for higher levels of mGFR (Figure 2). The differences between the upper and lower limits of agreement were 48.3 mL/min/1.73 m^2^ and 50.1 mL/min/1.73 m^2^ for the CKD-Epi and the aMDRD formula, respectively. However, the mean bias was negligible since it reflects the mean of over- and underestimation of individual mGFR values. The absolute differences between measured and estimated GFR values significantly increased (CKD-Epi: p<0.01, r = 0.248; aMDRD: p<0.001, r = 0.462) for increasing levels of baseline mGFR (Figure 3). Analyses considering separately subjects with mGFR at inclusion < or ≥70 mL/min/1.73 m^2^ (Figure 4) showed that the accuracy of both prediction formulas was poor in either group (Table 2).

**Table 2:** Performance of CKD-Epi and aMDRD equations in predicting GFR at inclusion in 111 ADPKD patients as a whole and ranked according to mGFR<70 (n = 45) and ≥70 (n = 66) mL/min/1.73 m^2^.

		Overall	mGFR<70	mGFR≥70
**CKD-Epi**	**Estimated GFR**	81.37±29.39∧	52.85±12.58	100.82±20.18∧
	**Bias**	2.81±12.32	0.85±7.99	4.15±14.46
	**Mean % Error**	3.84±15.74	2.15±14.91	4.98±16.30
	**Mean Absolute % Error**	12.50±10.24	11.94±9.01	12.88±11.06
	**Scatter**	7.01	4.69	9.88
	**Mean Absolute Differences**	9.58±8.20	6.26±4.96	11.84±9.18
	**Estimates within 10%**	51.35	51.11	51.52
	**Pearson Coefficient**	0.908	0.775	0.715
	**Lin Coefficient**	0.899	0.760	0.692
**aMDRD**	**Estimated GFR**	73.01±27.95*	47.82±10.39*	90.18±22.59°
	**Bias**	−5.55±12.79	−4.17±7.08	−6.49±15.51
	**Mean % Error**	−6.93±14.69	−7.23±13.01	−6.72±15.82
	**Mean Absolute % Error**	13.32±9.23	11.76±9.01	14.38±9.29
	**Scatter**	8.92	4.60	11.24
	**Mean Absolute Differences**	10.76±8.82	6.34±5.18	13.77±9.53
	**Estimates within 10%**	41.44	51.11	34.85
	**Pearson Coefficient**	0.891	0.770	0.729
	**Lin Coefficient**	0.872	0.712	0.672

Iohexol plasma clearance: overall: 78.56±26.70 mL/min/1.73 m^2^; GFR<70: 51.99±10.49 mL/min/1.73 m^2^; GFR≥70: 96.67±17.63 mL/min/1.73 m^2^.

Data are mean±SD or median.

Estimated GFR, Bias, Scatter and Mean Absolute Differences are in mL/min/1.73 m^2^.

* p<0.001;

° p<0.01;

∧ p<0.05 vs. iohexol plasma clearance.

### Relationships between measured and estimated GFR changes at one year vs. baseline

Measured and estimated one-year GFR data were available in 71 of the 111 included patients. Demography, clinical and laboratory characteristics at inclusion of patients with or without one-year outcome data were similar, with the exception of mGFR and serum creatinine levels (Table 1). Consistently with data in the whole study group, baseline mGFR values were significantly overestimated and underestimated by CKD-Epi and aMDRD formulas, respectively. At one year the difference between estimated and measured GFRs was still significant only when CKD-Epi estimates were considered (Table 3).

**Table 3:** Measured and estimated one-year GFR changes vs. baseline in 71 ADPKD patients as a whole and ranked according to mGFR<70 (n = 25) and ≥70 (n = 46) mL/min/1.73 m^2^.

		Overall	mGFR<70	mGFR≥70
**Iohexol**	**Baseline GFR**	83.13±27.52	52.64±10.28	99.69±18.03
	**One-Year GFR**	74.70±27.83	45.52±9.77	90.56±20.58
	**GFR Change**	−8.43±10.31	−7.13±7.51	−9.13±11.57
**CKD-Epi**	**Baseline GFR**	86.84±29.56∧	54.10±12.05	104.63±18.31∧
	**One-Year GFR**	81.84±32.41*	47.00±14.40	100.78±21.96*
	**GFR Change**	−4.99±8.96∧	−7.10±6.29	−3.85±10.00°
**aMDRD**	**Baseline GFR**	77.94±28.54°	48.74±9.70∧	93.81±22.04∧
	**One-Year GFR**	73.41±29.97	42.74±11.68	90.08±22.75
	**GFR Change**	−4.53±9.73∧	−6.00±5.46	−3.72±11.39∧

Data are in mL/min/1.73 m^2^; mean±SD.

* p<0.001;

° p<0.01;

∧ p<0.05 vs. iohexol plasma clearance.

**Figure 5:**
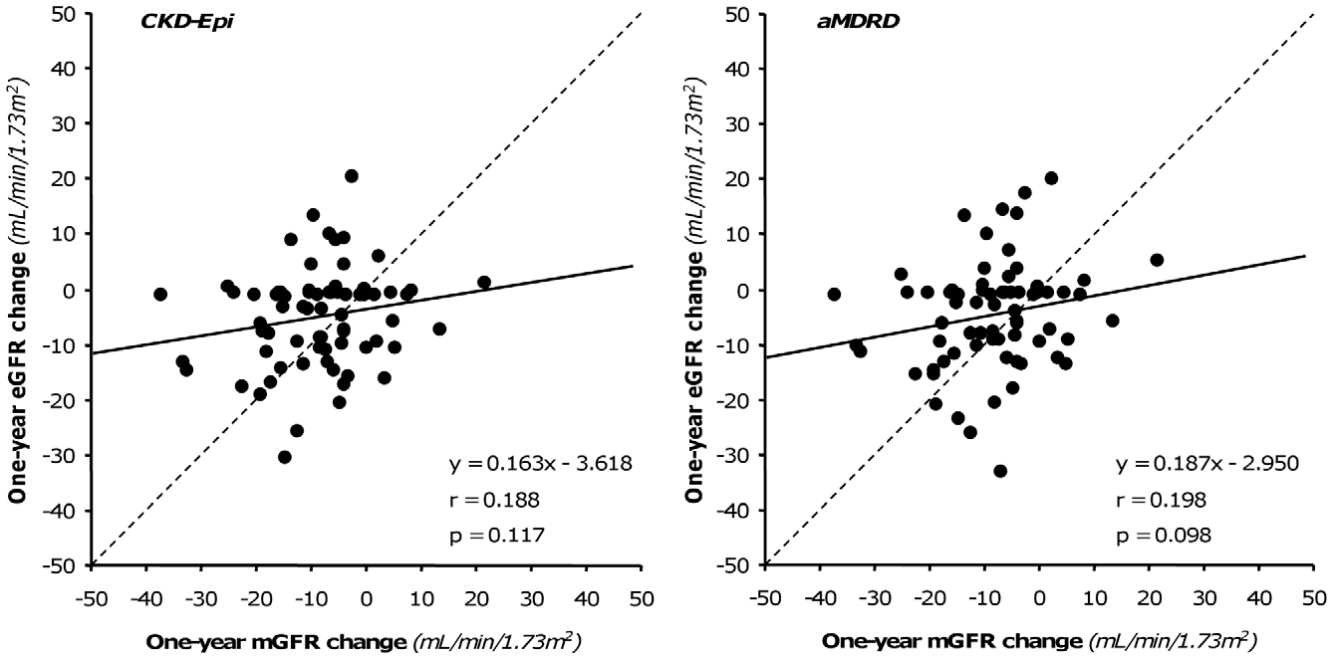
Relationship between measured and estimated 1-year GFR changes. Correlation between measured 1-year GFR changes vs. baseline and corresponding changes estimated by CKD-Epi (Left panel) and aMDRD (Right panel) formulas. Dot lines are identity lines; continuous lines are regression lines.

**Figure 6:**
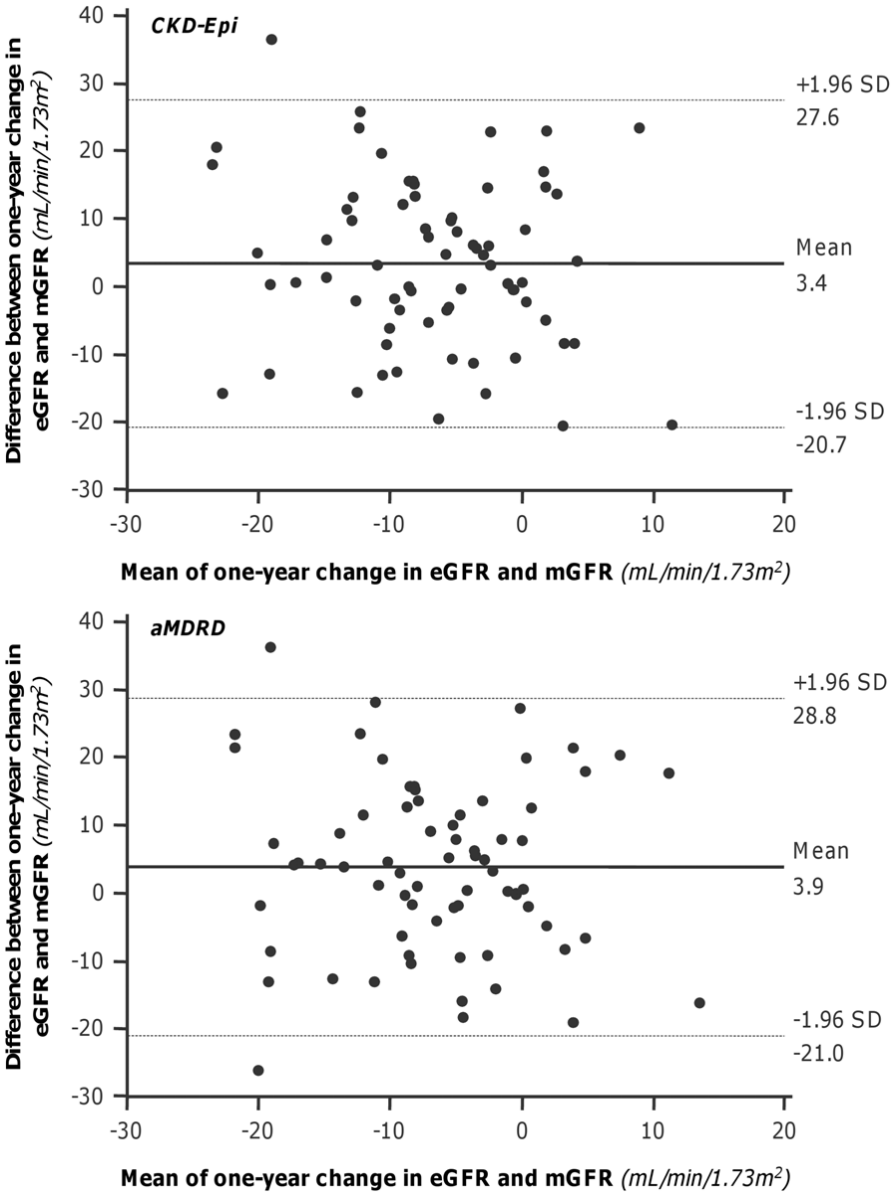
Bland-Altman plots of measured and estimated 1-year changes. Graphs show the agreement between estimated by CKD-Epi (Upper panel) or by aMDRD (Lower panel) formulas and corresponding measured 1-year GFR changes vs. baseline. Straight line and dashed lines indicate mean difference and 95% limits of agreement, respectively.

**Figure 7:**
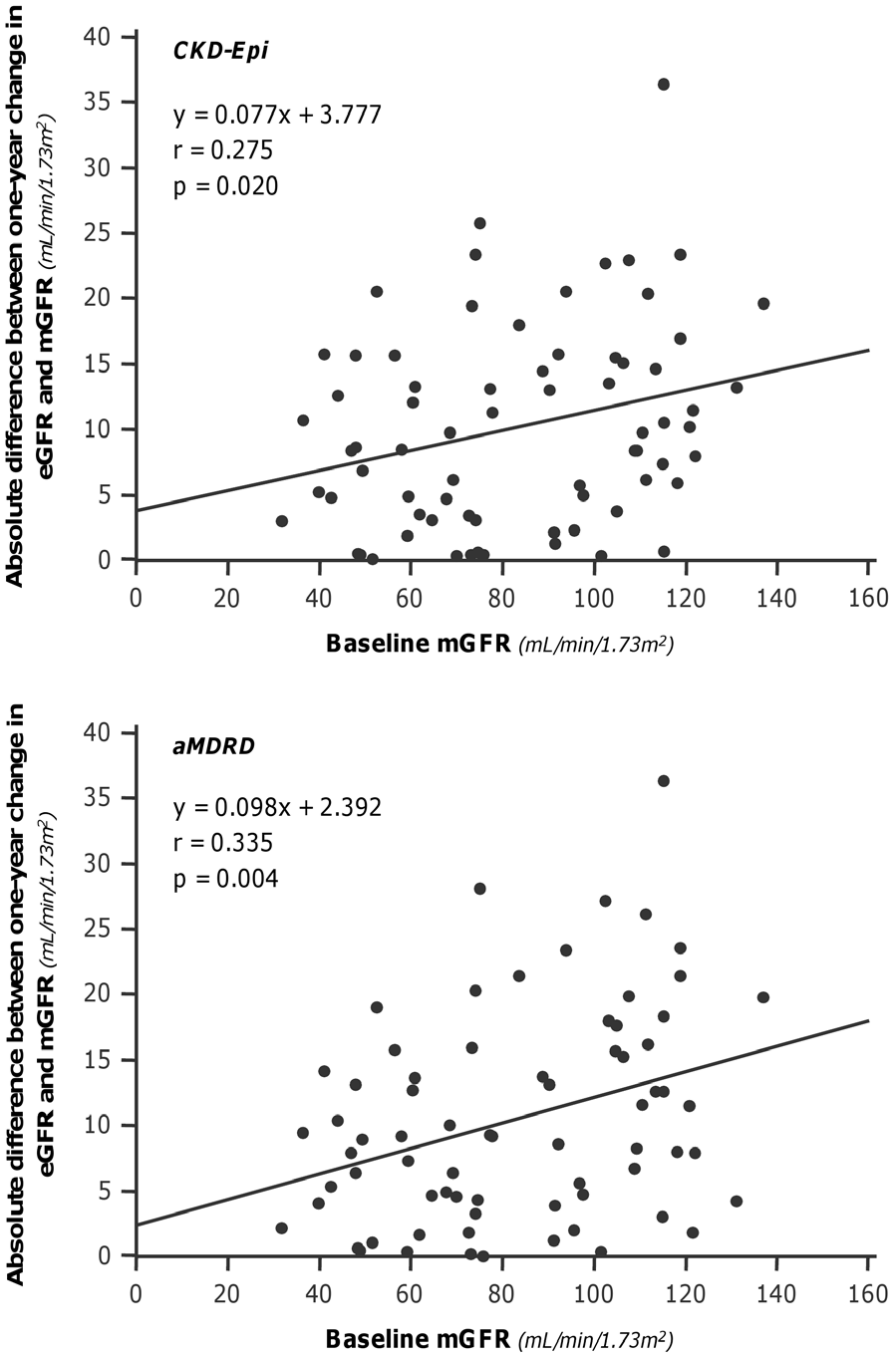
Absolute differences between measured and estimated 1-year GFR changes vs baseline measured GFR. The absolute differences between 1-year GFR changes for both CKD-Epi (Upper panel) and aMDRD (Lower panel) formulas significantly increase for increasing values of GFR. Continuous lines are regression lines.

**Figure 8:**
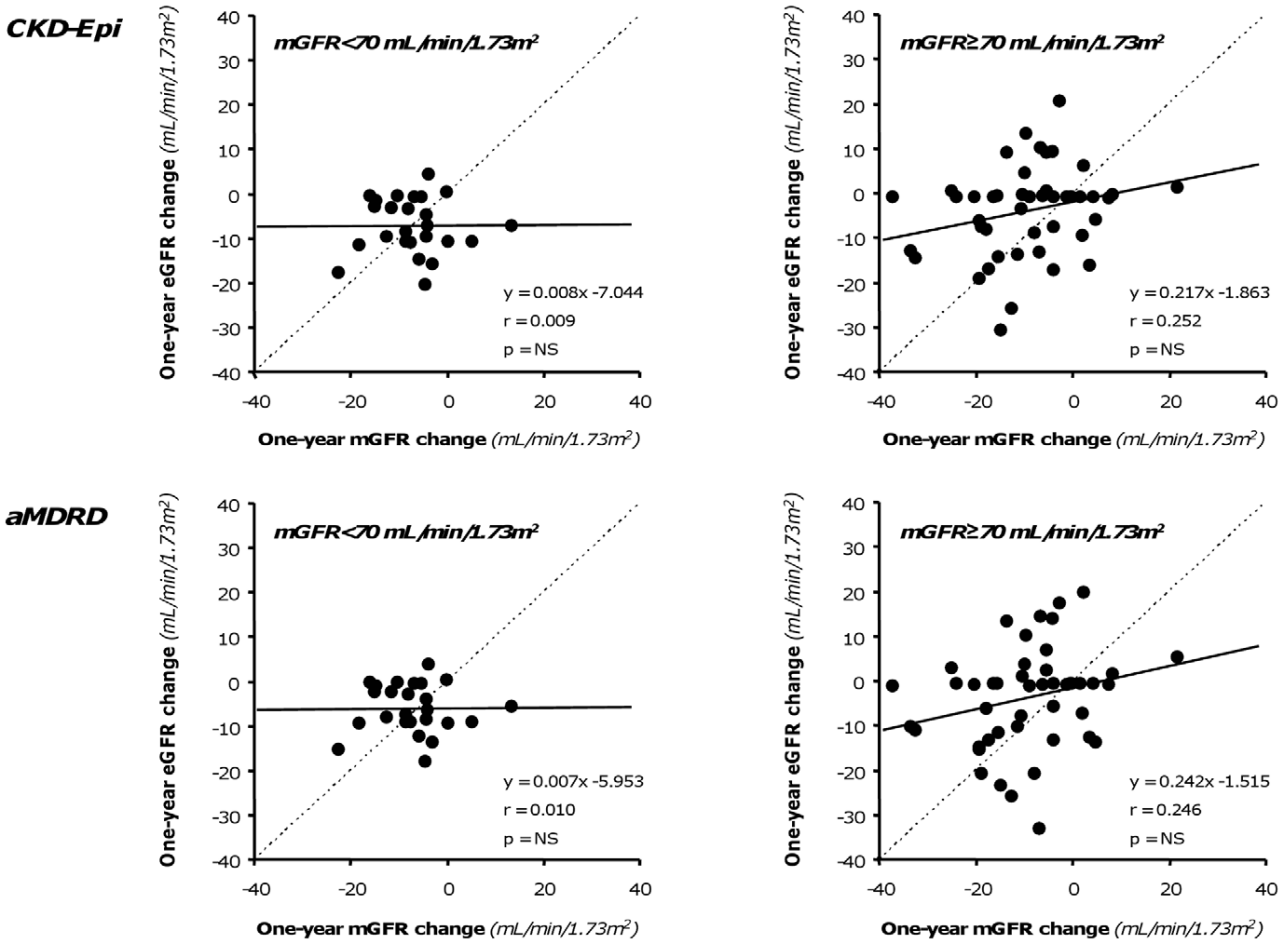
Relationship between 1-year GFR changes ranked according to renal function. Correlation between measured 1-year GFR changes vs. baseline and corresponding changes estimated by CKD-Epi (Upper panels) and aMDRD (Lower panels) formulas in patients with baseline GFR< or ≥70 mL/min/1.73 m^2^ considered separately (Left and Right panels, respectively). Dot lines are identity lines; continuous lines are regression lines.

Overall, at one-year, mGFR decreased by 8.4 mL/min/1.73 m^2^ vs. baseline, a reduction that CKD-Epi and aMDRD significantly underestimated by 59% and 53%, respectively (Table 3). Bias, mean percent errors and mean absolute percent errors of estimated vs. measured one-year GFR changes were similar with the two equations (Table S1). Only 8.57% and 5.71% of the CKD-Epi and aMDRD estimates deviated by less than 10% from actual values, respectively. The accuracy was poor for both estimates, although the percentage of acceptable estimates was slightly higher with the CKD-Epi than with the aMDRD formula. With both formulas, scatter and mean absolute differences between measured and estimated GFR changes approximated 10 mL/min/1.73 m^2^ (Table S1), a value that exceeded the 8.4 mL/min/1.73 m^2^ GFR change actually measured at one year (Table 3). No significant correlation was found between mGFR changes and changes estimated either by the CKD-Epi and the aMDRD formula (Figure 5). At Bland-Altman analyses, the performance of the two equations was similarly poor at any level of renal function changes (Figure 6). The differences between the upper and lower limits of agreement were 48.3 mL/min/1.73 m^2^ and 49.8 mL/min/1.73 m^2^ for the CKD-Epi and the aMDRD formula, respectively. The absolute differences between measured and estimated GFR changes significantly increased (CKD-Epi: p = 0.020 r = 0.275; aMDRD: p = 0.004, r = 0.335) for increasing levels of baseline mGFR (Figure 7). Actually, the analysis of the subgroups of subjects with mGFR at inclusion < or ≥70 mL/min/1.73 m^2^ showed that the accuracy in assessing GFR change by both CKD-Epi and aMDRD formulas was poorer for mGFR higher than 70 mL/min/1.73 m^2^ (Table 3). Nevertheless, as shown in Figure 8, even in subjects with mGFR<70 mL/min/1.73 m^2^ the extent of GFR changes predicted by both formulas was fully independent of actually measured changes. Consistently, in this subgroup of subjects estimates of one-year GFR changes based on CKD-Epi and aMDRD equations deviated with large percent errors from actual changes measured by iohexol plasma clearance (Table S1).

## Discussion

The key findings of our present analysis in a relatively large cohort of adult ADPKD patients who had their GFR values centrally measured by a gold standard procedure such as the iohexol plasma clearance technique [21] and at the same time estimated by the CKD-Epi and aMDRD prediction formulas, can be summarized in the following 3 points:

- GFR values estimated by the two formulas significantly correlated with measured GFRs. Data, however, were biased by a significant overestimation with the CKD-Epi and underestimation with the aMDRD formula. Moreover, there was a wide and unpredictable deviation of estimated data from measured values, with less than 50 percent of GFR values being predicted with an adequate accuracy by the two equations.
- One-year GFR changes estimated by both prediction formulas failed to correlate to any appreciable extent with measured changes. Moreover, data were biased by a systematic underestimation of measured GFR changes that averaged 50 percent with both formulas. Again, there was a wide and unpredictable deviation of estimated from measured GFR changes, with less than nine percent of GFR changes being reliably predicted by the two equations. Of note, deviations of estimated data even exceeded the actually measured GFR changes.
- Because of imprecise estimation of actual GFR values and unreliable prediction of GFR changes over time, both CKD-Epi and aMDRD equations fail to provide useful information in the setting of clinical trials aimed to test the effect of experimental treatments on progressive renal function loss in patients with ADPKD.

In a previous prospective analysis of ADPKD patients with baseline GFR>70 mL/min/1.73 m^2^, GFR slopes calculated on the basis of serial GFR measurements by iothalamate clearance better correlated with a series of baseline predictors of disease progression than GFR slopes calculated by using aMDRD and Cockcroft-Gault GFR estimates [36]. The above findings can be explained by the bias in calculating GFR slopes using creatinine-based prediction equations. Actually, other Authors have suggested that in early stages of CKD the variability in serum creatinine levels might reflect creatinine production related to muscle mass or protein intake more than glomerular filtration [36].

Our present data confirm that prediction formulas, including the CKD-Epi equation - not considered in previous studies - are far from accurate in estimating GFR and are fully unreliable in estimating GFR changes in subjects with ADPKD, and provide formal evidence that this limitation is independent of kidney function and applies also to individuals with more severe renal insufficiency. This is in harmony with cross-sectional data by Orskov and colleagues [37] showing that the performance of prediction formulas, including CKD-Epi and aMDRD in estimating renal function, was poor across a wide range of GFRs from CKD stage 1 to 5. Here we extend these data by providing the fully novel evidence that the CKD-Epi and aMDRD formulas do not allow any useful information to predict GFR changes over time, a limitation that, again, applies also to subjects with lower GFRs to start with. These findings are in line with previous observations in other population, such as in kidney transplant recipients, showing that predictive performance of GFR equations, including aMDRD and Cockcroft-Gault formulas, in detecting renal function changes over time was remarkably inferior to that of GFR measurements with iohexol plasma clearance [35]. On the other hand, the wide variability of GFR estimates we observed in our ADPKD patients might be explained by changes in tubular creatinine handling that could be specific to the disease. Creatinine accumulating into non-communicating cysts, in particular in those originating from proximal tubuli, cannot be excreted into urine [1] and might back-diffuse into the circulation. We speculate that this would induce serum creatinine changes that are independent of glomerular filtration and that might bias any GFR estimation based on serum creatinine levels.

As demonstrated in our present analyses, both underestimation and dispersion of data synergistically converge to decrease the power of statistical analyses aimed to demonstrate a treatment effect on GFR. In this perspective, failure to detect any, even marginal, correlation between measured and estimated GFR changes over one year follow-up, definitely challenged the reliability of any clinical trial using CKD-Epi and aMDRD equations to test the effects of experimental treatments in ADPKD [12], [13]. Similar considerations apply to the several prediction formulas developed over the last 40 years for GFR estimation that are flawed (even to a larger extent) by the same limitations described for the above equations.

In another perspective, an encouraging implication of the above findings is that the results of studies based on the use of prediction formulas cannot be taken to definitely discard the idea that mTOR inhibitors may be suitable for the treatment of ADPKD [6].

### Limitations and Strengths

The major limitation of our study was the *post-hoc* nature of an observational analysis of subjects included in trials originally designed for other purposes. Moreover, since longitudinal data were available only for a subgroup, GFR changes over time could be analyzed in a relatively small number of patients. Finally, the availability of only two sequential GFR measurements per patient did not allow comparative analyses between slopes of measured and estimated GFRs. Thus, our present findings can be considered as hypothesis generating and merit confirmation in ad hoc studies formally comparing GFR changes over time directly measured by gold standard procedures and indirectly estimated by using prediction formulas. A major strength was that all patients were monitored according to predefined and standardized guidelines and by using a standard procedure for GFR measurement largely applied to monitor renoprotective effects of given treatments on renal function in patients with CKD participating to clinical trials [38]–[42]. Iohexol plasma clearance also showed a good agreement with inulin renal clearance (the gold standard for renal function assessment) in subjects with different degree of renal function [21], [28], [29]. Consistently, GFR decline measured in our study patients was quite similar to that previously reported after the fourth decade of age in ADPKD patients prospectively monitored by serial GFR measurements by using the iothalamate plasma clearance technique [43].

Moreover, finding that no patients was on concomitant treatments known to affect creatinine tubular handling, avoided the confounding effect of GFR-independent changes in serum creatinine levels that might have further reduced the reliability of prediction formulas that use serum creatinine as an endogenous marker of glomerular filtration. Our present data also had a large external validity since selection criteria allowed identifying a study population which is representative of the average population of ADPKD patients who refer to a Nephrology Unit in every day clinical practice. Moreover, GFR estimates were based on serum creatinine levels measured in laboratories of different centers by using validated local procedures, which faithfully reflects how prediction formulas are routinely used in real life.

### Conclusions

In our present series of patients with ADPKD, independent of their kidney function, prediction formulas, including those that have been most recently implemented to improve the performance in GFR estimation [14] unreliably estimated actual GFR values and failed to detect their changes over time. Study findings suggest that these surrogate outcome variables are not appropriate to assess progression of ADPKD and response to treatment in research and clinics. Long-term, adequately powered clinical trials with direct measurement of kidney function by appropriate techniques may help better evaluating the efficacy of therapeutic strategies in this clinical setting, as well as in other chronic kidney diseases.

## Supporting Information

Table S1 Performance of CKD-Epi and aMDRD equations in predicting one-year GFR changes vs. baseline in 71 ADPKD patients as a whole and ranked according to mGFR<70 (n = 25) and ≥70 (n = 46) mL/min/1.73 m^2^.
(DOC)
