# Supplementary material for: Measuring and Estimating GFR and Treatment Effect in ADPKD Patients: Results and Implications of a Longitudinal Cohort Study
Source: PLoS One. 2012 Feb 28;7(2):e32533. doi: 10.1371/journal.pone.0032533 (PMC3291245; doi:10.1371/journal.pone.0032533)
Supplement: Table S1 — Performance of CKD-Epi and aMDRD equations in predicting one-year GFR changes vs. baseline in 71 ADPKD patients as a whole and ranked according to mGFR<70 (n = 25) and ≥70 (n = 46) mL/min/1.73 m2. (DOC) [file pone.0032533.s001.doc]

|  |  | **Overall** | **mGFR<70** | **mGFR≥70** |
| --- | --- | --- | --- | --- |
| **CKD-Epi** | **Bias** | 3.43±12.32 | 0.03±9.75 | 5.28±13.25 |
|  | **Mean % error** | -1575.21±12661.06 | -4292.21±21344.97 | -98.57±196.05 |
|  | **Mean Absolute % Error** | 1440.3±12677.34 | 4193.18±21365.46 | -55.84±212.56 |
|  | **Scatter** | 8.61 | 6.85 | 10.92 |
|  | **Mean Absolute Differences** | 10.16±7.69 | 7.86±5.54 | 11.41±8.42 |
|  | **Estimates within 10%** | 8.57 | 8.00 | 8.89 |
|  | **Pearson Coefficient** | 0.188 | 0.009 | 0.252 |
|  | **Lin Coefficient** | 0.175 | 0.009 | 0.222 |
| **aMDRD** | **Bias** | 3.90±12.70 | 1.12±9.24 | 5.41±14.10 |
|  | **Mean % error** | -1387.54±11134.68 | -3789.58±18768.05 | -82.08±224.82 |
|  | **Mean Absolute % Error** | 1270.02±11148.89 | 3683.67±18790.01 | -41.75±235.90 |
|  | **Scatter** | 9.14 | 7.31 | 11.55 |
|  | **Mean Absolute Differences** | 10.52±8.03 | 7.56±5.22 | 12.13±8.84 |
|  | **Estimates within 10%** | 5.71 | 8.00 | 4.44 |
|  | **Pearson Coefficient** | 0.198 | 0.001 | 0.246 |
|  | **Lin Coefficient** | 0.184 | 0.009 | 0.220 |

Data are mean±SD or median.

Bias, Scatter and Mean Absolute Differences are in mL/min/1.73m^2^
